# Genetic Determinants of Leisure-Time Physical Activity in the Hungarian General and Roma Populations

**DOI:** 10.3390/ijms24054566

**Published:** 2023-02-26

**Authors:** Péter Pikó, Éva Bácsné Bába, Zsigmond Kósa, János Sándor, Nóra Kovács, Zoltán Bács, Róza Ádány

**Affiliations:** 1 ELKH-DE Public Health Research Group, Department of Public Health and Epidemiology, Faculty of Medicine, University of Debrecen, 4032 Debrecen, Hungary; 2 Epidemiology and Surveillance Centre, Semmelweis University, 1085 Budapest, Hungary; 3 Institute of Sport Economics and Management, Faculty of Economics and Business, University of Debrecen, 4032 Debrecen, Hungary; 4 Department of Health Methodology and Public Health, Faculty of Health, University of Debrecen, 4400 Nyíregyháza, Hungary; 5 Department of Public Health and Epidemiology, Faculty of Medicine, University of Debrecen, 4032 Debrecen, Hungary; 6 Department of Accounting, Faculty of Economics and Business, University of Debrecen, 4032 Debrecen, Hungary; 7 Department of Public Health, Semmelweis University, 1089 Budapest, Hungary

**Keywords:** genetics, leisure-time physical activity, polygenic score, Roma population, Hungarian population

## Abstract

Leisure-time physical activity (LTPA) is one of the modifiable lifestyle factors that play an important role in the prevention of non-communicable (especially cardiovascular) diseases. Certain genetic factors predisposing to LTPA have been previously described, but their effects and applicability on different ethnicities are unknown. Our present study aims to investigate the genetic background of LTPA using seven single nucleotide polymorphisms (SNPs) in a sample of 330 individuals from the Hungarian general (HG) and 314 from the Roma population. The LTPA in general and three intensity categories of it (vigorous, moderate, and walking) were examined as binary outcome variables. Allele frequencies were determined, individual correlations of SNPs to LTPA, in general, were determined, and an optimized polygenetic score (oPGS) was created. Our results showed that the allele frequencies of four SNPs differed significantly between the two study groups. The C allele of rs10887741 showed a significant positive correlation with LTPA in general (OR = 1.48, 95% CI: 1.12–1.97; *p* = 0.006). Three SNPs (rs10887741, rs6022999, and rs7023003) were identified by the process of PGS optimization, whose cumulative effect shows a strong significant positive association with LTPA in general (OR = 1.40, 95% CI: 1.16–1.70; *p* < 0.001). The oPGS showed a significantly lower value in the Roma population compared with the HG population (oPGS_Roma_: 2.19 ± SD: 0.99 vs. oPGS_HG_: 2.70 ± SD: 1.06; *p* < 0.001). In conclusion, the coexistence of genetic factors that encourage leisure-time physical activity shows a more unfavorable picture among Roma, which may indirectly contribute to their poor health status.

## 1. Introduction

Urbanization and the spread of technological innovations [1], as well as the restrictions during the COVID-19 pandemic [2], have contributed greatly to the sudden decline in physical activity in recent years. Today, physical inactivity is a severe public health problem, as the prevalence of a sedentary lifestyle among adults is increasing worldwide [3]. Physical inactivity is an important preventable risk factor for non-communicable diseases [3,4]. Leisure-time physical activity (LTPA) is a well-known modifiable lifestyle factor associated with a wide range of cardiometabolic outcomes, including obesity, hypertension, type 2 diabetes, metabolic syndrome, and cardiovascular diseases in general [5]. 

Various psychological, biological, social, and environmental factors affecting leisure-time physical activity have been investigated and identified [6,7,8]. Demographic and health variables associated with levels of physical activity include sex, age, education, and body mass index (BMI) [9]. Recognizing the demographic, environmental, and social determinants of physical activity among adults is important for designing effective intervention strategies to promote it [10]. Several studies have shown that some determinants, such as age, higher educational attainment, and higher income, are associated with increased participation in LTPA for some groups [11,12]. Despite this knowledge and continued efforts to encourage physical activity, in most developed countries prevalence remains low and participation rates for women are consistently lower than for men [13].

Leisure-time physical activity is influenced by a combination of several factors, of which genetic heritability is estimated by studies to be between 30% and 52% [7]. A study published in 2009 involving 1644 unrelated Dutch and 978 Americans of European ancestry found that the heritability of leisure-time physical activity behavior is explained by a large number of genetic variants with small individual effect sizes [14]. A 2014 study by Kim et al. [15] in a sample of 8842 Koreans found similar results, with no significant association of single nucleotide polymorphisms (SNPs) with LTPA at the individual level, but 59 SNPs (in 76 genes) were identified using multiple SNP bootstrap analysis. LTPA varies between different ethnic groups [16,17], which can be partly explained by the environmental and lifestyle characteristics mentioned above, but differences in the genetic background cannot be excluded [18].

The Roma comprise the largest minority (10–12 million) in Europe, originating from the Punjab region of northern India as a nomadic people, and arriving in Europe between the eighth and tenth centuries A.D. [19,20]. The health status of Roma is generally much worse than that of the general population, regardless of the country where they live [21,22,23]. Studies on their health are almost exclusively descriptive on the prevalence of certain diseases [24], particularly infectious [25,26] and certain genetics-related diseases/conditions [27,28,29], and health determinants [20,30,31,32,33,34] and cardiovascular risk factors [35,36,37,38,39,40], while comprehensive exploratory studies are lacking.

Although the association between the very unfavorable socioeconomic circumstances and unfavorable health status [31,41,42] of Roma is evident, it seems that the differences observed in comparison with the general populations cannot be explained solely by their poorer socioeconomic characteristics [30,43]. Recent studies on the genetic background of increased risk of various non-communicable diseases among them [44,45,46,47,48] further support the hypothesis that their health status is determined by the complex interactions of health-related genetic and non-genetic factors.

The physical activity of Roma has not been well characterized; data on physical activity from the 2011 cross-sectional, population-based HepaMeta survey in Slovakia showed that LTPA was significantly lower among Roma women than among non-Roma [49]. Regarding the health risk behavior of Roma adolescents in segregated settlements in Slovakia compared to non-Roma, the differences were not statistically significant, except for the significantly higher rate of physical inactivity among Roma women [50]. The results of a complex health survey carried out by our research team in 2018 [51] are similar to those reported in Slovakia, in that while there is no significant difference in LTPA between Roma and non-Roma men, Roma women were found to have significantly lower levels than non-Roma.

An article published in 2019 [52], comparing the physical activity levels of two Roma subgroups (Gabor and Băieși) and non-Roma groups in Romania, found that both Roma subgroups had significantly lower levels of daily physical activity (with gender differences). In addition, both Roma subgroups were less active than non-Roma in sports and gardening.

Previous studies have shown that there is a marked difference in the genetic background of the Roma and majority population in cardiometabolic health-related factors such as high-density lipoprotein cholesterol (HDL-C) levels [44,46], type 2 diabetes [53,54], obesity [55,56,57,58], venous thrombosis [45,59,60], hypertension [61], smoking [62], and alcohol consumption [63,64].

Given that physical activity is to a large extent genetically determined and that there are differences in leisure-time physical activity between the Hungarian general and the Roma population, the question arises whether these differences are not due, at least partly, to different genetic backgrounds resulting from their different origins.

Our study aims to investigate whether LTPA is also genetically determined in the Hungarian general and Roma populations and how this contributes to the lower LTPA among Roma using previously identified polymorphisms that promote LTPA.

## 2. Results

### 2.1. Characteristics of the Study Populations by Sex

No significant differences were found in mean age, abdominal circumference, and BMI between the two study populations by sex. In the Roma population, the proportion with lower levels of education were significantly higher, and the proportion of people traveling by vehicle was significantly lower (HG_men_ = 81.4% vs. Roma_men_ = 26.6%, *p* < 0.001; HG_women_ = 68.1% vs. Roma_women_ = 24.7%, *p* < 0.001) in both sexes. See Table 1 for more details.

In general, the proportion of people who did LTPA was not significantly different between the two study populations for either sex.

For men in the Hungarian general population, the proportion of participants with LTPA of vigorous (HG: 39.3% vs. Roma: 12.7%, *p* < 0.001) and moderate (HG: 32.4% vs. Roma: 10.2%, *p* = 0.016) intensity was significantly higher than among Roma, while the proportions of people walking in leisure time did not differ significantly between the two study populations (HG: 53.8% vs. Roma: 54.4%, *p* = 0.927).

For women, similarly to men, a significantly higher proportion of the Hungarian general population did vigorous (HG: 32.4% vs. Roma: 10.2%, *p* < 0.001) or moderate (HG: 42.7% vs. Roma: 25.1%, *p* < 0.001) intensity of LTPA and there was no significant difference in walking (HG: 62.7% vs. Roma: 57.9%, *p* = 0.316). See Table 2 for more details.

For men, a significant difference in metabolic equivalent of task minutes per week (MET-min/week) values was only measured in the LTPA category of vigorous-intensity between the two study populations (HG: 792.0 vs. Roma: 323.1, *p* < 0.001). Among women in the Hungarian general population, the average MET-min/week values for LTPA in general (HG: 1357.6 vs. Roma: 1052.5, *p* = 0.028) as well as in vigorous (HG: 647.2 vs. 311.3, *p* < 0.001), moderate (HG: 559.9 vs. 407.3, *p* = 0.003), and walking (HG: 463.2 vs. 247.73, *p* = 0.011) subdomains were significantly higher than among Roma. See Table 3 for more details.

### 2.2. Results of Linkage Disequilibrium (LD), Hardy-Weinberg Equilibrium (HWE), and Power Analyses and Comparison of Genotype Distribution between Sample Populations

In LD analysis of ten SNPs, there was no linkage between SNPs. For three SNPs (rs12405556, rs429358, and rs6092090), significant deviations from HWE were measured and these SNPs were excluded from further analysis.

For four (rs10252228, rs12612420, rs459465, and rs10887741) of the seven SNPs included in the study, a significant allele frequency difference was found between the Hungarian general and Roma populations and the statistical power varied between 0.147 and 0.985. See Supplementary Table S1 for more details.

### 2.3. The Result of the Association of SNPs with LTPA of Different Intensities

Only the C allele of rs10887741 showed a significant positive association with LTPA in general (odds ratio (OR) = 1.48, 95% CI: 1.12–1.97, *p* = 0.006), but none of the seven SNPs included in the study showed a significant association with any intensity category. For more details see Table 4.

### 2.4. Calculation and Comparison of Optimized Polygenic Score (PGS) for LTPA in the Hungarian General and Roma Populations

The PGS optimization process tests the cumulative effect of SNPs by starting with the SNP showing the strongest association with LTPA (rs10887741: OR = 1.48, *p* = 0.006) and in decreasing order to the weakest one (rs459465: OR = 1.01, *p* = 0.967). During the process, rs6022999 and rs7023003 increased the strength of association of optimized polygenic score (oPGS) with LTPA in general. The remaining four SNPs (rs12612420, rs10252228, rs8097348, and rs459465) did not increase the strength of association and were therefore excluded from further analysis. See more details in Supplementary Table S2.

Based on univariate analysis, oPGS showed a significant positive correlation with the LTPA in general (OR = 1.40, 95% CI: 1.17–1.68; *p* < 0.001) and in intensity categories of vigorous (OR = 1.32, 95% CI: 1.09–1.59; *p* = 0.004) and moderate (OR = 1.23, 95% CI: 1.04–1.46; *p* = 0.013). After adjusting for confounders (ethnicity, age, waist circumference, BMI, education, and driving), the association remained significant only for the LTPA in general (OR = 1.40, 95% CI: 1.16–1.70, *p* < 0.001). For more details see Table 5.

In the groups defined based on oPGS values, we examined how the METS-min/week values changed for LTPA in general and its intensity categories and conducted a trend analysis. With the increase in oPRS, there was a significant upward trend in LTPA expressed in MET-min/week in general (*p* for trend = 0.002) as well as in the vigorous intensity category (*p* for trend = 0.015). The moderate (*p* for trend = 0.028) and walking (*p* for trend = 0.019) intensity categories showed no significant correlation with the oPGS categories after the test correction. For more details see Table 6.

We also examined how the oPGS values are related to the weekly frequency of LTPA, i.e., the average number of days with at least 10 min that a person engages in leisure-time physical activity in general and its intensity categories. In this case, as in the MET-min/week results, there is a significant trend between the increase in oPGS values and the number of days per week of leisure-time physical activity in general (*p* for trend = 0.001), as well as for the vigorous (*p* for trend = 0.003), moderate (*p* for trend = 0.014), and walking (*p* for trend = 0.009) intensity categories. For more details see Supplementary Table S3.

**Figure 1:**
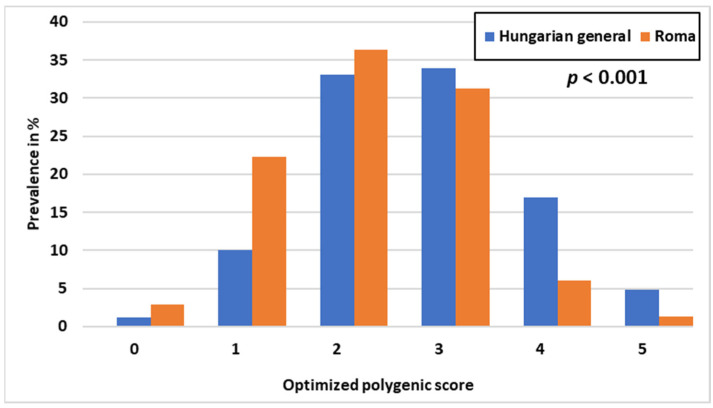
Distribution of participants by optimized polygenic score values for leisure-time physical activity in the Hungarian general and Roma populations.

The distribution of oPGS differed significantly between the two study populations (oPGS_Roma_: 2.19 ± SD:0.99 vs. oPGS_HG_: 2.70 ± SD:1.06; *p* < 0.001). A strong rightward shift (to the higher values) is observed for the HG population compared with the Roma. See Figure 1 for more details.

## 3. Discussion

LTPA is low in both the Hungarian general and Roma populations [51], which may be due to the influence of genetic background [7,65] in addition to known environmental and lifestyle factors [66]. The aim of the present study is to test this hypothesized genetic effect and, if it exists, to compare its magnitude between the Hungarian general and Roma populations.

Based on a systematic literature search, ten SNPs were selected to investigate the genetic background of LTPA. Of the ten SNPs selected, three were excluded based on HWE, while four of the remaining seven had significant allele frequency differences between the two populations. When examining the individual effects of SNPs, only the C allele of rs10887741 showed a significant association with LTPA. PGS optimization identified three SNPs for which the combined effect showed a strong positive significant association with LTPA in general, and oPGS categories are significantly correlated with an increasing trend in MET-min/week values as well as with the frequency of LTPA in general and vigorous-intensity categories.

The distribution of the populations by oPGS shows a significant shift to the right in the Hungarian general population compared with the Roma population. This finding suggests that the Hungarian general population has a higher genetic predisposition to doing leisure-time physical activity compared to the Roma.

The rs10887741 polymorphism in the 3′-phosphoadenosine 5′-phosphosulfate synthase 2 (*PAPSS2*) gene showed the strongest individual association with LTPA in general. The enzyme encoded by the *PAPSS2* gene is involved in the sulfation of many molecules in addition to glycosaminoglycans. At present, the mechanisms by which the *PAPSS2* gene affects participation in leisure-time physical activity are not known, but mutations in it cause spondyloepimetaphyseal dysplasia, a disease characterized by short stature and limbs in both mice and humans [67]. A study on siblings found a correlation between the 10q23 region harboring the *PAPSS2* gene and maximum physical performance [68]. This further supports the hypothesis that physical fitness may be an important determinant of leisure-time physical activity behavior [69].

The rs6022999 SNP is located in the *CYP24A1* (Cytochrome P450 family 24 subfamily A member 1) gene, whose protein product is responsible for the conversion of vitamin D into its physiologically inactive form. Vitamin D is essential for proper muscle function [70,71], and polymorphisms of the vitamin D receptor in humans are associated with altered muscle strength regardless of sex [72]; these changes are likely to affect levels of physical activity.

The rs7023003 is located in an intergenic region between the *RN7SK* and *SLC44A1* genes. This SNP showed the strongest association with LTPA in a Korean study but still did not reach genome-wide association study significance [15]. Its significant association with LTPA was not confirmed in the Japanese population [73]. Currently, no research has investigated its role in LTPA through direct or indirect processes.

The importance of understanding the genetic reasons behind differences in individual (leisure-time) physical activity is supported by recently published articles. Doherty and colleagues investigated the genetic background of physical activity and sleep duration (both were based on measured data) in 91,105 individuals registered in the UK Biobank [74]. They successfully identified 14 significant loci (seven novel–five for LTPA and two for sleeping) accounting for 0.06% of physical activity and 0.39% of sleep duration. They found that the heritability was higher in women than in men for general activity (23% vs. 20%, *p* = 1.5 × 10^−4^) and sedentary behavior (18% vs. 15%, *p* = 9.7 × 10^−4^). Klimentidis et al. [75] also investigated UK Biobank samples and identified ten loci with a significant (*p* < 5 × 10^−9^) effect on all physical activity measures. Of these, the variant rs429358 in the *APOE* gene (which was excluded from our study due to its deviation from HWE) was most strongly associated with moderate to vigorous physical activity. A GWAS study by Wang et al. [76] successfully identified a combination of 99 genetic variants associated with self-reported moderate to vigorous leisure-time physical activity, leisure-time screen time and/or sedentary behavior at work. Results summarized in a review article by De Geus in 2023 [77] support the general opinion that genetic factors strongly contribute to physical activity either self-reported or measured by accelerometer. The heritability of physical activity was found to be approximately 43% across the lifespan. It has also been shown that a polygenic score based on genetic variants influencing PA (which we also use) could help to improve the success of targeted interventions.

This study has its strengths and limitations. First, the correct identification of ethnicity is a common challenge in studies like ours [78]. Roma ethnicity was determined solely through self-identification, and consequently, there may be Roma individuals in the Hungarian general population, so the effect of ethnic differences in the study may be underestimated. Another limitation is that individuals who are above 65 years of age were not included in the study. Owing to a lack of information on gene-gene and gene-environment interactions, epigenetic factors, and structural variants, we did not consider them in our analysis. In the current study, ten SNPs related to LTPA were included for the calculation of oPGS. Theoretically, incorporating a larger number of SNPs may further improve the predictive ability of the PGS model. Nonetheless, adding many SNPs into the PGS model does not necessarily lead to a better predictive ability, as could be seen in the optimization process. Despite the limitations of the study, it should be emphasized that this is the first study to examine the possible genetic causes of the unfavorable level of leisure-time physical activity in the Roma population in comparison with that in the Hungarian general population.

In conclusion, the present study demonstrates that the differences in the prevalence of different intensity categories of LTPA between the Hungarian general and Roma populations can be partly explained by genetic causes.

## 4. Materials and Methods

### 4.1. Sample Populations and Questionnaire-Based Interviews

Data used in our present study were obtained in a cross-sectional three-pillar (i.e., questionnaire-based, physical examination, and laboratory examination) complex (i.e., health behavior and examination) survey carried out in 2018. Sampling and data collection are described in detail elsewhere [79].

Briefly, the Hungarian general (HG) and Roma sample populations were recruited from two counties (Hajdú-Bihar and Szabolcs-Szatmár-Bereg) in Northeast Hungary, the area where the representation of Roma is the highest and where most segregated Roma colonies are located. First, twenty-five colonies, and then from each colony 20 households, were randomly selected and one person (aged 20–64) from each household was invited to participate in the survey. Participants’ ethnicity was determined by self-declaration. The Hungarian general population included randomly selected individuals aged 20 to 64 years, living in private households in the same counties, and registered with general practitioners. From each of the 20 randomly selected GP practices, 25 randomly selected individuals were invited to participate in the study. The planned sample size of the survey was 500 persons per population, but the final study sample, for the present study, was reduced to 797 (410 HG and 387 Roma) after excluding individuals with incomplete records.

The main part of the questionnaire used in the complex health survey was the European Health Interview Survey wave 2 (EHIS 2) questionnaire [80], which consists of four modules: (a) health status, (b) health care utilization, (c) determinants of health, and (d) socioeconomic variables. The EHIS 2 questionnaire has been extended with some additional sets of questions, including the long version of the International Physical Activity Questionnaire (IPAQ) to measure physical activity by domains and dimensions. Only activities performed for at least ten minutes during the last seven days were recorded in the questionnaire.

### 4.2. Characterization of LTPA by Sub-Domains and Intensity Categories

The IPAQ measures time spent in different areas (sub-domains): (1) work, (2) transport, (3) home and gardening, and (4) leisure in three intensity categories (walking, moderate-intensity activity, and vigorous-intensity activity). For details on calculating physical activity levels, see elsewhere [51].

Briefly, individuals who (regardless of intensity category) performed any form of physical activity in their leisure time (strictly outside working hours) were included in the group of people who performed LTPA.

The three intensity categories of LTPA are based on the form of exercise performed:

Walking: leisure walks outside working hours;

Moderate: non-strenuous exercise (outside of walking in leisure time), such as light cycling, swimming, table tennis, jogging, etc.;

Vigorous: strenuous leisure time activity, such as: running, fast cycling, swimming, dancing, aerobics, etc.

In addition, LTPA intensity was quantified as weekly metabolic equivalent task minutes (MET-min/week) based on participants’ responses according to the IPAQ scoring protocol [81]. Total minutes over the last seven days spent on different types of LTPA were defined for each individual to create MET-min/week scores for activity sub-domains, and average values were calculated for both sample populations by sex.

### 4.3. DNA Extraction, SNP Selection, Genotyping, Testing Hardy-Weinberg Equilibrium, and Linkage Disequilibrium

DNA was extracted from EDTA-anticoagulated blood samples using the MagNA Pure LC system (Roche Diagnostics, Basel, Switzerland) following the manufacturer’s instructions.

Using online search engines such as PubMed, Ensemble, and HuGE navigator, a systematic literature search was conducted to identify SNPs statistically significantly associated with LTPA. The search time frame related to the present study was until 5 August 2019. Keywords and their combinations used in the search: leisure time physical activity, recreational physical activity, genetics, genome-wide association study (GWAS), candidate gene, genotype. In the selection of SNPs, particular attention was focused on the results of the three GWAS [14,15,73] and a candidate gene study [82], which were the most relevant in this field.

The literature search identified a total of ten SNPs, and these were genotyped using the MassARRAY platform (Sequenom Inc., San Diego, CA, USA) with iPLEX Gold chemistry in the Mutation Analysis Core Facility (MAF) of the Karolinska University Hospital, Sweden. The MAF conducted validation, concordance analysis, and quality control according to their protocols. The Hardy-Weinberg Equilibrium (HWE) and linkage disequilibrium (LD) structure of the genotyped SNPs were calculated by Haploview software (version 4.2; Broad Institute; Cambridge, MA, USA).

### 4.4. Calculation and Optimization of the Polygenic Score

Individuals with any missing SNP genotypes were excluded from further analyses; thus, 330 participants from the HG sample and 314 Roma individuals were included in genotype analysis. In the PGS calculation, each person was assigned a score based on the number of effect alleles carried. The effect allele was considered to be the allele that promotes LTPA. Homozygous effect alleles were considered as “2”, heterozygotes as “1”, and genotypes with no effect allele were considered as “0”.

By using these codes, a simple count score was calculated as described by Equation (1), in which *Gi* is the number of the effect alleles for the *i*th SNP. This model sums up all alleles over all loci as a summary score, assuming that all alleles have the same effect in direction and size.

(1) $$\;GRS=\sum_{i=1}^{I}Gi$$

The polygenic model optimization procedure aimed to select SNPs (identified in the systematic literature search) that had a strong association with LTPA in both study populations. For PGS optimization, adjusted logistic regression analyses (for age, ethnicity, sex, education, traveling by vehicle, BMI, and waist circumference) were used, and these analyses were also performed on a combined sample of the two populations.

The SNPs were tested in ascending order of *p*-value, in which process each SNP was inserted into the statistical model one by one, starting from the SNP with the strongest association (with the lowest *p*-value), and the association between oPGS and LTPA was examined after each insertion.

SNPs were selected and used for final oPGS only if they increased the strength of association of oPGS (decreased *p*-value and increased Cox-Snell R-squared value) with LTPA. SNPs that did not affect or weaken the model’s association, i.e., increased the *p*-value and decreased the R-squared, were excluded from further analyses.

Genetic predisposition categories were formed based on the population distribution of oPGS, four groups were created, and trend analysis was used to examine the association of these groups with LTPA in general and its intensity categories.

### 4.5. Statistical Analysis

The χ^2^ test was used to compare the differences between nonquantitative variables and to examine the HWE of genotyped SNPs. Statistical power for each SNP was calculated by using the Online Sample Size Estimator (OSSE) online tool (http://osse.bii.a-star.edu.sg/calculation1.php) (accessed on 10 January 2023). The Shapiro–Wilk test was used to examine whether the quantitative variables were normally distributed or not, and, if necessary, Templeton’s two-step method was considered to transform the non-normal variables into normal ones [83]. The Mann–Whitney U test was used to assess the distribution of age, waist circumference, BMI, oPGS, and MET-min/week between the study populations.

Multiple logistic regression analyses were used to determine the association between genetic factors (individual SNPs and oPGS) and LTPA. All regression analyses were conducted using a model adjusted for relevant factors (e.g., age, ethnicity, sex, education, vehicle travel, BMI, and waist circumference). The Jonckheere–Terpstra trend test [84] was used to analyze the trend of association between oPGS categories and MET-min/week values. Ethnicity was used as a covariate when the two populations were combined and examined together. Statistical analyses were performed using IBM Statistical Package for the Social Sciences (SPSS) version 26 (Armonk, NY, USA). For multiple statistical analyses (all calculations involving the oPGS), the Bonferroni correction method was used (the conventional *p*-value of 0.05 was divided by the number of independent polymorphisms).

### 4.6. Ethics Declarations

Informed consent was recorded for all subjects who were included in the study. The survey was conducted under the conditions set out in the Declaration of Helsinki and the protocol was approved by the Ethical Committee of the Hungarian Scientific Council on Health (61327-2017/EKU).

## Figures and Tables

**Table 1:** Characteristics of study populations by sex.

		Men			Women		
		Hungarian General (*n* = 145)	Roma (*n* = 79)	*p*-Value	Hungarian General (*n* = 185)	Roma (*n* = 235)	*p*-Value
		Mean (95% CI)			Mean (95% CI)		
Age (years)		44.73 (42.85–46.61)	45.68 (42.69–48.67)	0.373	43.43 (41.50–45.36)	41.64 (40.13–43.16)	0.143
Waist circumference (cm)		98.52 (96.42–100.61)	97.41 (93.51–101.30)	0.721	94.43 (92.06–96.81)	93.52 (91.43–95.61)	0.773
BMI (kg/m^2^)		27.33 (26.61–28.05)	27.68 (26.35–29.02)	0.861	27.24 (26.36–28.12)	27.47 (26.56–28.38)	0.994
		Prevalence in % (95% CI)		*p*-value	Prevalence in % (95% CI)		*p*-value
Education	Primary	17.93 (12.35–24.78)	92.41 (85.02–96.77)	<0.001 *	21.08 (15.68–27.38)	85.53 (80.61–89.58)	<0.001 *
	Secondary	63.45 (55.41–70.96)	7.59 (3.23–14.98)		58.92 (51.74–65.82)	13.62 (9.69–18.44)	
	Higher	17.93 (12.35–24.78)	0.00		19.46 (14.25–25.61)	0.43 (0.05–1.97)	
	Missing data	0.69 (0.07–3.18)	0.00		0.54 (0.06–2.50)	0.43 (0.05–1.97)	
Using a vehicle for traveling		81.38 (74.46–87.06)	26.58 (17.81–37.04)	<0.001 *	68.11 (61.15–74.50)	24.68 (19.50–30.48)	<0.001 *

* *p* < 0.05; 95% CI: 95% confidence interval.

**Table 2:** The proportion of people doing any type of leisure-time physical activity (LTPA) by different intensity categories and sex in the Hungarian general and Roma populations.

	Men			Women		
	Hungarian General (*n* = 145)	Roma (*n* = 79)	*p*-Value	Hungarian General (*n* = 185)	Roma (*n* = 235)	*p*-Value
	Prevalence in % (95% CI)			Prevalence in % (95% CI)		
LTPA in general	72.41 (64.75–79.19)	67.09 (56.26–76.69)	0.404	73.51 (66.83–79.47)	70.64 (64.59–76.18)	0.515
	Prevalence in % (95% CI)		*p*-value	Prevalence in % (95% CI)		*p*-value
Vigorous	39.31 (31.64–47.41)	12.66 (6.70–21.29)	<0.001 *	32.43 (26.00–39.41)	10.21 (6.83–14.57)	<0.001 *
Moderate	49.66 (41.59–57.73	32.91 (23.31–43.74)	0.016 *	42.70 (35.73–49.90)	25.11 (19.89–30.93)	<0.001 *
Walking	53.79 (45.67–61.77)	54.43 (43.45–65.09)	0.927	62.70 (55.58–69.43)	57.87 (51.49–64.06)	0.316

*: *p* < 0.05; 95% CI: 95% confidence interval.

**Table 3:** The average MET-min/week values for leisure-time physical activity (LTPA) in general and by different intensity subdomains in the Hungarian general and Roma populations by sex.

	Men			Women		
	Hungarian General (*n* = 145)	Roma (*n* = 79)	*p*-Value	Hungarian General (*n* = 185)	Roma (*n* = 235)	*p*-Value
	Average MET-Min/Week (95% CI)			Average MET-Min/Week (95% CI)		
LTPA in general	1443.3 (1169.9–1716.7)	1152.0 (757.6–1546.4)	0.198	1357.6 (1114.7–1600.5)	1052.5 (845.6–1259.4)	0.028 *
	Average MET-min/week (95% CI)		*p*-value	Average MET-min/week (95% CI)		*p*-value
Vigorous	792.0 (635.1–948.8)	323.1 (163.5–482.6)	<0.001 *	647.2 (517.8–776.5)	262.4 (182.8–342.0)	<0.001 *
Moderate	660.3 (527.0–793.5)	592.2 (353.6–830.8)	0.225	559.9 (441.5–678.4)	407.3 (284.5–530.0)	0.003 *
Walking	372.8 (276.2–469.3)	227.1 (137.0–317.2)	0.165	463.2 (377.7–548.7)	311.3 (249.3–373.4)	0.011 *

*: *p* < 0.05; MET-min/week: metabolic equivalent of task minutes per week; 95% CI: 95% confidence interval.

**Table 4:** Association of SNPs with general leisure-time physical activity (LTPA) and its different intensity categories.

SNP (Effect Allele) Chromosome Position ^£^	LTPA in General	Vigorous	Moderate	Walking
	Odds Ratio (95% CI)			
rs10252228 (G) 7:34900427	1.09 (0.85–1.40) *p* = 0.429	1.07 (0.81–1.42) *p* = 0.618	1.15 (0.91–1.46) *p* = 0.242	1.18 (0.94–1.48) *p* = 0.155
rs12612420 (G) 2:200293399	1.09 (0.82–1.45) *p* = 0.542	0.95 (0,68–1.33) *p* = 0.753	0.97 (0.74–1.29) *p* = 0.858	1.00 (0.77–1.30) *p* = 0.985
rs7023003 (G) 9:105118389	1.25 (0.88–1.76) *p* = 0.212	1.15 (0.80–1.65) *p* = 0.443	1.15 (0.84–1.56) *p* = 0.388	1.15 (0.84–1.56) *p* = 0.379
rs459465 (G) 20:54806483	(0.72–1.40) *p* = 0.967	0.97 (0.68–1.38) *p* = 0.852	0.89 (0.66–1.21) *p* = 0.453	1.20 (0.89–1.61) *p* = 0.235
rs6022999 (A) 20:54171474	1.29 (0.98–1.70) *p* = 0.065	0.94 (0.68–1.30) *p* = 0.715	0.90 (0.69–1.18) *p* = 0.468	1.20 (0.93–1.55) *p* = 0.151
rs10887741 (C) 10:87683553	1.48 (1.12–1.97) *p* = 0.006 *	1.25 (0.93–1.68) *p* = 0.140	1.23 (0.95–1.59) *p* = 0.108	1.15 (0.89–1.47) *p* = 0.278
rs8097348 (A) 18:1595020	1.06 (0.79–1.42) *p* = 0.697	0.99 (0.70–1.38) *p* = 0.946	1.10 (0.83–1.47) *p* = 0.497	1.20 (0.91–1.57) *p* = 0.194

^£^ Based on Genome Reference Consortium Human Build 38 patch (GRCh38). *: *p* < 0.05; 95% CI: 95% confidence interval.

**Table 5:** The association of the optimized polygenic score based on three SNPs with leisure-time physical activity in general and with different intensity categories is based on univariate and multivariate regression analyses. In multivariate analyses, adjustments were made for ethnicity, sex, age, waist circumference, BMI, education, and travel by vehicle.

	Univariate		Multivariate	
	Odds Ratio (95% CI)	*p*-Value	Odds Ratio (95% CI)	*p*-Value
LTPA in general	1.40 (1.17–1.68)	<0.001 **	1.40 (1.16–1.70)	<0.001 **
	Odds ratio (95% CI)	*p*-value	Odds ratio (95% CI)	*p*-value
Vigorous	1.32 (1.09–1.59)	0.004 **	1.13 (0.91–1.39)	0.260
Moderate	1.23 (1.04–1.46)	0.013 **	1.09 (0.92–1.31)	0.314
Walking	1.16 (0.99–1.37)	0.064	1.19 (1.00–1.41)	0.043 *

*: *p* < 0.05; **: significant results after test correction (*p* < 0.017); 95% CI: 95% confidence interval.

**Table 6:** Trend analysis of MET-min/week value change for leisure-time physical activity (LTPA) in general and in different intensity categories in relation to optimized polygenic score (oPGS) values.

	oPGS (0–1) *n* = 116	oPGS (2) *n* = 223	oPGS (3) *n* = 210	oPGS (4–5) *n* = 95	*p* for Trend
	Average MET-Min/Week (95% CI)				
LTPA in general	990.95 (667.72–1314.18)	1081.17 (862.39–1299.95)	1350.10 (1139.66–1560.54)	1679.29 (1326.17–2032.41)	<0.001 **
	Average MET-min/week (95% CI)				*p* for trend
Vigorous	409.69 (268.93–550.44)	465.02 (360.08–569.96)	496.15 (381.98–610.33)	698.74 (511.50–885.98)	0.015 **
Moderate	511.92 (340.70–683.15)	455.95 (341.93–569.97)	499.42 (381.78–617.06)	798.77 (592.92–1004.62)	0.028 *
Walking	303.53 (208.41–398.65)	310.40 (238.07–387.72)	426.93 (355.05–498.81)	387.02 (277.45–496.59)	0.019 *

*: *p* < 0.05; **: significant results after test correction (*p* < 0.017); MET-min/week: metabolic equivalent of task minutes per week; 95% CI: 95% confidence interval.

## Data Availability

Data available on request due to privacy or ethical concerns.

## Supplementary Materials

The following supporting information can be downloaded at: https://www.mdpi.com/article/10.3390/ijms24054566/s1.

## References

[1] Woessner M.N., Tacey A., Levinger-Limor A., Parker A.G., Levinger P., Levinger I. (2021). The Evolution of Technology and Physical Inactivity: The Good, the Bad, and the Way Forward. Front. Public Health.

[2] Tison G.H., Barrios J., Avram R., Kuhar P., Bostjancic B., Marcus G.M., Pletcher M.J., Olgin J.E. (2022). Worldwide physical activity trends since COVID-19 onset. Lancet Glob. Health.

[3] Lee I.M., Shiroma E.J., Lobelo F., Puska P., Blair S.N., Katzmarzyk P.T., Lancet Physical Activity Series Working G. (2012). Effect of physical inactivity on major non-communicable diseases worldwide: An analysis of burden of disease and life expectancy. Lancet.

[4] World Health Organization (2003). Health and Development through Physical Activity and Sport.

[5] Norman A., Bellocco R., Vaida F., Wolk A. (2002). Total physical activity in relation to age, body mass, health and other factors in a cohort of Swedish men. Int. J. Obes. Relat. Metab. Disord..

[6] Molina-Garcia J., Castillo I., Queralt A. (2011). Leisure-time physical activity and psychological well-being in university students. Psychol. Rep..

[7] Aaltonen S., Kujala U.M., Kaprio J. (2014). Factors behind leisure-time physical activity behavior based on Finnish twin studies: The role of genetic and environmental influences and the role of motives. Biomed. Res. Int..

[8] Starr M.C. (2018). Environment perception and leisure-time physical activity in Portuguese high school students. Prev. Med. Rep..

[9] Hallal P.C., Victora C.G., Wells J.C., Lima R.C. (2003). Physical inactivity: Prevalence and associated variables in Brazilian adults. Med. Sci. Sports Exerc..

[10] Kawabata M., Chua K.L., Chatzisarantis N.L.D. (2018). A school-based intervention program in promoting leisure-time physical activity: Trial protocol. BMC Public Health.

[11] Lobaszewski J., Przewozniak K., Zatonska K., Wojtyla A., Bylina J., Manczuk M., Zatonski W.A. (2011). Patterns of leisure time physical activity and its determinants among a sample of adults from Kielce region, Poland—The ‘PONS’ study. Ann. Agric. Environ. Med..

[12] Momenan A.A., Delshad M., Mirmiran P., Ghanbarian A., Azizi F. (2011). Leisure Time Physical Activity and Its Determinants among Adults in Tehran: Tehran Lipid and Glucose Study. Int. J. Prev. Med..

[13] Rutten A., Ziemainz H., Schena F., Stahl T., Stiggelbout M., Auweele Y.V., Vuillemin A., Welshman J. (2003). Using different physical activity measurements in eight European countries. Results of the European Physical Activity Surveillance System (EUPASS) time series survey. Public Health Nutr..

[14] De Moor M.H., Liu Y.J., Boomsma D.I., Li J., Hamilton J.J., Hottenga J.J., Levy S., Liu X.G., Pei Y.F., Posthuma D. (2009). Genome-wide association study of exercise behavior in Dutch and American adults. Med. Sci. Sports Exerc..

[15] Kim J., Kim J., Min H., Oh S., Kim Y., Lee A.H., Park T. (2014). Joint identification of genetic variants for physical activity in Korean population. Int. J. Mol. Sci..

[16] de Munter J.S., Agyemang C., van Valkengoed I.G., Bhopal R., Zaninotto P., Nazroo J., Kunst A.E., Stronks K. (2013). Cross national study of leisure-time physical activity in Dutch and English populations with ethnic group comparisons. Eur. J. Public Health.

[17] Du L., Hong F., Luo P., Wang Z., Zeng Q., Guan H., Liu H., Yuan Z., Xu D., Nie F. (2022). Patterns and demographic correlates of domain-specific physical activities and their associations with dyslipidaemia in China: A multiethnic cohort study. BMJ Open.

[18] Aasdahl L., Nilsen T.I.L., Meisingset I., Nordstoga A.L., Evensen K.A.I., Paulsen J., Mork P.J., Skarpsno E.S. (2021). Genetic variants related to physical activity or sedentary behaviour: A systematic review. Int. J. Behav. Nutr. Phys. Act..

[19] Martinez-Cruz B., Mendizabal I., Harmant C., de Pablo R., Ioana M., Angelicheva D., Kouvatsi A., Makukh H., Netea M.G., Pamjav H. (2016). Origins, admixture and founder lineages in European Roma. Eur. J. Hum. Genet..

[20] European Commission (2014). Health Status of the Roma Population. Data Collection in the Member States of the European Union.

[21] Macejova Z., Kristian P., Janicko M., Halanova M., Drazilova S., Antolova D., Marekova M., Pella D., Madarasova-Geckova A., Jarcuska P. (2020). The Roma Population Living in Segregated Settlements in Eastern Slovakia Has a Higher Prevalence of Metabolic Syndrome, Kidney Disease, Viral Hepatitis B and E, and Some Parasitic Diseases Compared to the Majority Population. Int. J. Environ. Res. Public Health.

[22] Anthonj C., Setty K.E., Ezbakhe F., Manga M., Hoeser C. (2020). A systematic review of water, sanitation and hygiene among Roma communities in Europe: Situation analysis, cultural context, and obstacles to improvement. Int. J. Hyg. Environ. Health.

[23] van Dijk J.P. (2019). Roma health: Do we know enough?. Int. J. Public Health.

[24] Vincze F., Foldvari A., Palinkas A., Sipos V., Janka E.A., Adany R., Sandor J. (2019). Prevalence of Chronic Diseases and Activity-Limiting Disability among Roma and Non-Roma People: A Cross-Sectional, Census-Based Investigation. Int. J. Environ. Res. Public Health.

[25] Drazilova S., Kristian P., Janicko M., Halanova M., Safcak D., Dorcakova P.D., Marekova M., Pella D., Madarasova-Geckova A., Jarcuska P. (2020). What is the Role of the Horizontal Transmission of Hepatitis B Virus Infection in Young Adult and Middle-Aged Roma Population Living in the Settlements in East Slovakia?. Int. J. Environ. Res. Public Health.

[26] Tombat K., van Dijk J.P. (2020). Roma Health: An Overview of Communicable Diseases in Eastern and Central Europe. Int. J. Environ. Res. Public Health.

[27] Stiburkova B., Bohata J., Pavelcova K., Tasic V., Plaseska-Karanfilska D., Cho S.K., Potocnakova L., Saligova J. (2021). Renal Hypouricemia 1: Rare Disorder as Common Disease in Eastern Slovakia Roma Population. Biomedicines.

[28] Kocova M., Anastasovska V., Petlichkovski A., Falhammar H. (2021). First insights into the genetics of 21-hydroxylase deficiency in the Roma population. Clin. Endocrinol..

[29] Ivanov I.S., Azmanov D.N., Ivanova M.B., Chamova T., Pacheva I.H., Panova M.V., Song S., Morar B., Yordanova R.V., Galabova F.K. (2014). Founder p.Arg 446* mutation in the PDHX gene explains over half of cases with congenital lactic acidosis in Roma children. Mol. Genet. Metab..

[30] Voko Z., Csepe P., Nemeth R., Kosa K., Kosa Z., Szeles G., Adany R. (2009). Does socioeconomic status fully mediate the effect of ethnicity on the health of Roma people in Hungary?. J. Epidemiol. Community Health.

[31] Janevic T., Jankovic J., Bradley E. (2012). Socioeconomic position, gender, and inequalities in self-rated health between Roma and non-Roma in Serbia. Int. J. Public Health.

[32] Sandor J., Kosa Z., Boruzs K., Boros J., Tokaji I., McKee M., Adany R. (2017). The decade of Roma Inclusion: Did it make a difference to health and use of health care services?. Int. J. Public Health.

[33] Petraki I., Kalpourtzi N., Terzidis A., Gavana M., Vantarakis A., Rachiotis G., Karakosta A., Sypsa V., Touloumi G., Grp H.S. (2021). Living in Roma Settlements in Greece: Self-Perceived Health Status, Chronic Diseases and Associated Social Determinants of Health. Int. J. Environ. Res. Public Health.

[34] Orton L., de Cuevas R.A., Stojanovski K., Gamella J.F., Greenfields M., La Parra D., Marcu O., Matras Y., Donert C., Frost D. (2019). Roma populations and health inequalities: A new perspective. Int. J. Hum. Rights Hea.

[35] Babinska I., Veselska Z.D., Bobakova D., Pella D., Panico S., Reijneveld S.A., Jarcuska P., Jarcuska P., Zezula I., Geckova A.M. (2013). Is the cardiovascular risk profile of people living in Roma settlements worse in comparison with the majority population in Slovakia?. Int. J. Public Health.

[36] Zeljko H.M., Skaric-Juric T., Narancic N.S., Baresic A., Tomas Z., Petranovic M.Z., Milicic J., Salihovic M.P., Janicijevic B. (2013). Age trends in prevalence of cardiovascular risk factors in Roma minority population of Croatia. Econ. Hum. Biol..

[37] Piko P., Kosa Z., Sandor J., Adany R. (2021). Comparative risk assessment for the development of cardiovascular diseases in the Hungarian general and Roma population. Sci. Rep..

[38] Pallayova M., Brenisin M., Putrya A., Vrsko M., Drazilova S., Janicko M., Marekova M., Pella D., Geckova A.M., Urdzik P. (2020). Roma Ethnicity and Sex-Specific Associations of Serum Uric Acid with Cardiometabolic and Hepatorenal Health Factors in Eastern Slovakian Population: The HepaMeta Study. Int. J. Environ. Res. Public Health.

[39] Petranovic M.Z., Rizzieri A.E., Sivaraj D., Narancic N.S., Skaric-Juric T., Celinscak A., Markovic A.S., Salihovic M.P., Kalaszi J., Kalaszi M. (2021). CVD Risk Factors in the Ukrainian Roma and Meta-Analysis of Their Prevalence in Roma Populations Worldwide. J. Pers. Med..

[40] Hubacek J.A., Sedova L., Olisarova V., Adamkova V., Tothova V. (2021). Increased prevalence of the CVD-associated ANRIL allele in the Roma/Gypsy population in comparison with the majority Czech population. Genet. Mol. Biol..

[41] Sarvary A., Kosa Z., Javorne R.E., Gyulai A., Takacs P., Sandor J., Sarvary A., Nemeth A., Halmai R., Adany R. (2019). Socioeconomic status, health related behaviour, and self-rated health of children living in Roma settlements in Hungary. Cent Eur. J. Public Health.

[42] La Parra-Casado D., Mosquera P.A., Vives-Cases C., San Sebastian M. (2018). Socioeconomic Inequalities in the Use of Healthcare Services: Comparison between the Roma and General Populations in Spain. Int. J. Environ. Res. Public Health.

[43] Duval L., Wolff F.C., McKee M., Roberts B. (2016). The Roma vaccination gap: Evidence from twelve countries in Central and South-East Europe. Vaccine.

[44] Piko P., Fiatal S., Kosa Z., Sandor J., Adany R. (2017). Genetic factors exist behind the high prevalence of reduced high-density lipoprotein cholesterol levels in the Roma population. Atherosclerosis.

[45] Fiatal S., Piko P., Kosa Z., Sandor J., Adany R. (2019). Genetic profiling revealed an increased risk of venous thrombosis in the Hungarian Roma population. Thromb. Res..

[46] Piko P., Fiatal S., Werissa N.A., Bekele B.B., Racz G., Kosa Z., Sandor J., Adany R. (2020). The Effect of Haplotypes in the CETP and LIPC Genes on the Triglycerides to HDL-C Ratio and Its Components in the Roma and Hungarian General Populations. Genes.

[47] Sipeky C., Weber A., Szabo M., Melegh B.I., Janicsek I., Tarlos G., Szabo I., Sumegi K., Melegh B. (2013). High prevalence of CYP2C19*2 allele in Roma samples: Study on Roma and Hungarian population samples with review of the literature. Mol. Biol. Rep..

[48] Font-Porterias N., Gimenez A., Carballo-Mesa A., Calafell F., Comas D. (2021). Admixture Has Shaped Romani Genetic Diversity in Clinically Relevant Variants. Front. Genet..

[49] Babinska I., Geckova A.M., Jarcuska P., Pella D., Marekova M., Stefkova G., Veselska Z.D., HepaMeta T. (2014). Does the population living in Roma settlements differ in physical activity, smoking and alcohol consumption from the majority population in Slovakia?. Cent Eur. J. Public Health.

[50] Kolarcik P., Geckova A.M., Orosova O., van Dijk J.P., Reijneveld S.A. (2010). Predictors of health-endangering behaviour among Roma and non-Roma adolescents in Slovakia by gender. J. Epidemiol. Community Health.

[51] Bacsne Baba E., Piko P., Muller A., Rathonyi G., Balogh P., Kosa Z., Kovacs N., Sandor J., Adany R., Bacs Z. (2022). Physical Activity Pattern Characterized by Domains and Dimensions of the Roma Population in Comparison with That of the General Population in Northeast Hungary. Int. J. Environ. Res. Public Health.

[52] Szabó M.I., Balázs A., Máté B., Kelemen P. (2019). Low Level of Physical Activity in Two Roma Subgroups Compared to Non-Roma Population in Niraj Valley, Transylvania. J. Interdiscip. Med..

[53] Werissa N.A., Piko P., Fiatal S., Kosa Z., Sandor J., Adany R. (2019). SNP-Based Genetic Risk Score Modeling Suggests No Increased Genetic Susceptibility of the Roma Population to Type 2 Diabetes Mellitus. Genes.

[54] Hubacek J.A., Sedova L., Olisarova V., Adamkova V., Tothova V. (2020). Different prevalence of T2DM risk alleles in Roma population in comparison with the majority Czech population. Mol. Genet. Genom. Med..

[55] Nagy K., Fiatal S., Sandor J., Adany R. (2017). Distinct Penetrance of Obesity-Associated Susceptibility Alleles in the Hungarian General and Roma Populations. Obes. Facts.

[56] Zeljko H.M., Skaric-Juric T., Narancic N.S., Tomas Z., Baresic A., Salihovic M.P., Starcevic B., Janicijevic B. (2011). E2 allele of the apolipoprotein E gene polymorphism is predictive for obesity status in Roma minority population of Croatia. Lipids Health Dis..

[57] Poveda A., Ibanez M.E., Rebato E. (2012). Heritability and genetic correlations of obesity-related phenotypes among Roma people. Ann. Hum. Biol..

[58] Macekova S., Bernasovsky I., Gabrikova D., Bozikova A., Bernasovska J., Boronova I., Behulova R., Svickova P., Petrejcikova E., Sotak M. (2012). Association of the FTO rs9939609 polymorphism with obesity in Roma/Gypsy population. Am. J. Phys. Anthropol..

[59] Natae S.F., Kosa Z., Sandor J., Merzah M.A., Bereczky Z., Piko P., Adany R., Fiatal S. (2021). The Higher Prevalence of Venous Thromboembolism in the Hungarian Roma Population Could Be Due to Elevated Genetic Risk and Stronger Gene-Environmental Interactions. Front. Cardiovasc. Med..

[60] Bereczky Z., Gindele R., Fiatal S., Speker M., Miklos T., Balogh L., Mezei Z., Szabo Z., Adany R.A. (2021). Age and Origin of the Founder Antithrombin Budapest 3 (p.Leu131Phe) Mutation; Its High Prevalence in the Roma Population and Its Association With Cardiovascular Diseases. Front. Cardiovasc. Med..

[61] Staff P.O. (2021). Correction: The genetic risk for hypertension is lower among the Hungarian Roma population compared to the general population. PLoS ONE.

[62] Merzah M., Kosa Z., Sandor J., Natae S., Piko P., Adany R., Fiatal S. (2021). Roma Socioeconomic Status Has a Higher Impact on Smoking Behaviour than Genetic Susceptibility. Int. J. Environ. Res. Public Health.

[63] Dioszegi J., Fiatal S., Toth R., Moravcsik-Kornyicki A., Kosa Z., Sandor J., McKee M., Adany R. (2017). Distribution Characteristics and Combined Effect of Polymorphisms Affecting Alcohol Consumption Behaviour in the Hungarian General and Roma Populations. Alcohol Alcohol.

[64] Hubacek J.A., Sedova L., Olisarova V., Adamkova V., Adamek V., Tothova V. (2018). Distribution of Adh1b Genotypes Predisposed to Enhanced Alcohol Consumption in the Czech Roma/Gypsy Population. Cent Eur. J. Public Health.

[65] Lin X., Chan K.K., Huang Y.T., Luo X.I., Liang L., Wilson J., Correa A., Levy D., Liu S. (2018). Genetic Determinants for Leisure-Time Physical Activity. Med. Sci. Sports Exerc..

[66] Choi J., Lee M., Lee J.K., Kang D., Choi J.Y. (2017). Correlates associated with participation in physical activity among adults: A systematic review of reviews and update. BMC Public Health.

[67] Faiyaz ul Haque M., King L.M., Krakow D., Cantor R.M., Rusiniak M.E., Swank R.T., Superti-Furga A., Haque S., Abbas H., Ahmad W. (1998). Mutations in orthologous genes in human spondyloepimetaphyseal dysplasia and the brachymorphic mouse. Nat. Genet..

[68] Rico-Sanz J., Rankinen T., Rice T., Leon A.S., Skinner J.S., Wilmore J.H., Rao D.C., Bouchard C. (2004). Quantitative trait loci for maximal exercise capacity phenotypes and their responses to training in the HERITAGE Family Study. Physiol. Genom..

[69] de Geus E.J.C. (2021). A genetic perspective on the association between exercise and mental health in the era of genome-wide association studies. Ment. Health Phys. Act..

[70] Endo I., Inoue D., Mitsui T., Umaki Y., Akaike M., Yoshizawa T., Kato S., Matsumoto T. (2003). Deletion of vitamin D receptor gene in mice results in abnormal skeletal muscle development with deregulated expression of myoregulatory transcription factors. Endocrinology.

[71] Pfeifer M., Begerow B., Minne H.W. (2002). Vitamin D and muscle function. Osteoporos. Int..

[72] Windelinckx A., De Mars G., Beunen G., Aerssens J., Delecluse C., Lefevre J., Thomis M.A. (2007). Polymorphisms in the vitamin D receptor gene are associated with muscle strength in men and women. Osteoporos. Int..

[73] Hara M., Hachiya T., Sutoh Y., Matsuo K., Nishida Y., Shimanoe C., Tanaka K., Shimizu A., Ohnaka K., Kawaguchi T. (2018). Genomewide Association Study of Leisure-Time Exercise Behavior in Japanese Adults. Med. Sci. Sports Exerc..

[74] Doherty A., Smith-Byrne K., Ferreira T., Holmes M.V., Holmes C., Pulit S.L., Lindgren C.M. (2018). GWAS identifies 14 loci for device-measured physical activity and sleep duration. Nat. Commun..

[75] Klimentidis Y.C., Raichlen D.A., Bea J., Garcia D.O., Wineinger N.E., Mandarino L.J., Alexander G.E., Chen Z., Going S.B. (2018). Genome-wide association study of habitual physical activity in over 377,000 UK Biobank participants identifies multiple variants including CADM2 and APOE. Int. J. Obes..

[76] Wang Z., Emmerich A., Pillon N.J., Moore T., Hemerich D., Cornelis M.C., Mazzaferro E., Broos S., Ahluwalia T.S., Bartz T.M. (2022). Genome-wide association analyses of physical activity and sedentary behavior provide insights into underlying mechanisms and roles in disease prevention. Nat. Genet..

[77] De Geus E.J.C. (2023). Genetic Pathways Underlying Individual Differences in Regular Physical Activity. Exerc. Sport Sci. Rev..

[78] Janka E.A., Vincze F., Ádány R., Sándor J. (2018). Is the Definition of Roma an Important Matter? The Parallel Application of Self and External Classification of Ethnicity in a Population-Based Health Interview Survey. Int. J. Environ. Res. Public Health.

[79] Adany R., Piko P., Fiatal S., Kosa Z., Sandor J., Biro E., Kosa K., Paragh G., Bacsne Baba E., Veres-Balajti I. (2020). Prevalence of Insulin Resistance in the Hungarian General and Roma Populations as Defined by Using Data Generated in a Complex Health (Interview and Examination) Survey. Int. J. Environ. Res. Public Health.

[80] European Commission (2013). European Health Interview Survey (EHIS Wave 2) Methodological Manual.

[81] International Physical Activity Questionnaire (2005). Guidelines for Data Processing and Analysis of the International Physical Activity Questionnaire (IPAQ)—Short and Long Forms.

[82] Kostrzewa E., Brandys M.K., van Lith H.A., Kas M.J. (2015). A candidate syntenic genetic locus is associated with voluntary exercise levels in mice and humans. Behav. Brain Res..

[83] Templeton G.F. (2011). A two-step approach for transforming continuous variables to normal: Implications and recommendations for IS research. Commun. Assoc. Inf. Syst..

[84] Gaur A. (2017). A class of k-sample distribution-free tests for location against ordered alternatives. Commun. Stat. Theor. Methods.

