# Supplementary material for: Genetic Determinants of Leisure-Time Physical Activity in the Hungarian General and Roma Populations
Source: Int J Mol Sci. 2023 Feb 26;24(5):4566. doi: 10.3390/ijms24054566 (PMC10003125; doi:10.3390/ijms24054566)
Supplement: Supplementary file 1 [file ijms-24-04566-s001.zip › Supplementary Table S1.pdf]

Supplementary Table S1. Genotype distribution in the Hungarian general and Roma populations and the results of Hardy-Weinberg equilibrium (HWE) analysis by single nucleotide polymorphisms (SNPs) tested.

SNP (rs number)	Gene	Genotype	Hungarian general (Prevalence in %)	Roma (Prevalence in %)	*p*-value for allele frequency comparison	*p*-value for HWE
rs10252228	*NPSR1, DPY19L1*	**G/G**	21.5	12.1	0.004*	0.082
		A/**G**	43.6	44.9		
		A/A	34.9	43.0		
rs10887741	*PAPSS2*	T/T	37.8	57.0	<0.001*	0.530
		T/**C**	45.5	38.9		
		**C/C**	16.7	4.1		
rs12612420	*SPATS2L*	A/A	5.8	7.6	<0.001*	0.257
		A/**G**	31.2	45.9		
		**G/G**	63.0	46.5		
rs459465	Intergenic	**G/G**	56.6	78.7	<0.001*	0.510
		**G**/A	38.2	19.1		
		A/A	5.2	2.2		
rs6022999	*CYP24A1*	G/G	5.2	9.6	0.065	0.110
		**A**/G	33.9	35.6		
		**A/A**	60.9	54.8		
rs7023003	*RN7SK, SLC44A* (intergenic)	**G/G**	4.5	1.9	0.081	0.130
		A/**G**	26.4	22.9		
		A/A	69.1	75.2		
rs8097348	*C18orf2*	G/G	4.2	5.7	0.332	0.822
		**A**/G	33.0	36.9		
		**A/A**	62.7	57.3		
Excluded due to significant deviation from HWE						
rs12405556	*LEPR*	**G/G**	62.1	70.7	0.063	0.008*
		**G**/T	31.2	24.8		
		T/T	6.7	4.5		
rs429358	*APOE*	C/C	1.5	5.1	<0.001*	0.024*
		**T**/C	16.4	28.7		
		**T/T**	82.1	66.2		
rs6092090	Intergenic	C/C	3.4	1.0	0.084	0.011*
		**T**/C	32.4	36.3		
		**T/T**	64.2	62.7		

LTPA-promoting alleles are highlighted in bold; *: p <0.05.
