# Supplementary material for: Genetic Determinants of Leisure-Time Physical Activity in the Hungarian General and Roma Populations
Source: Int J Mol Sci. 2023 Feb 26;24(5):4566. doi: 10.3390/ijms24054566 (PMC10003125; doi:10.3390/ijms24054566)
Supplement: Supplementary file 1 [file ijms-24-04566-s001.zip › Supplementary Table S2.pdf]

Supplementary Table S2. List of single nucleotide polymorphisms (SNPs) in order of strength of association with leisure-time physical activity (LTPA) in general (from the SNP with the strongest to the SNP with the weakest effect) used for optimized polygenetic score optimization and their effect on the strength of association (expressed as the *p*-value) with LTPA in general by adjusted logistic regression models.

SNP (effect allele)	OR (95%CI)	*p*-value	Cox & Snell R^2	Include/exclude
rs10887741 (C)	1.48 (1.12 – 1.97)	0.006	0.038	Included
rs6022999 (A)	1.39 (1.11 – 1.70)	0.001	0.042	Included
rs7023003 (G)	1.37 (1.15 – 1.64)	<0.001	0.045	Included
rs12612420 (G)	1.29 (1.11 – 1.50)	0.001	0.043	Excluded
rs10252228 (G)	1.27 (1.10 – 1.46)	0.001	0.042	Excluded
rs8097348 (A)	1.29 (1.10 – 1.50)	0.001	0.042	Excluded
rs459465 (G)	1.29 (1.10 – 1.52)	0.002	0.042	Excluded

OR: odds ratio; 95%CI: 95% confidence interval.
