# Supplementary material for: Genetic Determinants of Leisure-Time Physical Activity in the Hungarian General and Roma Populations
Source: Int J Mol Sci. 2023 Feb 26;24(5):4566. doi: 10.3390/ijms24054566 (PMC10003125; doi:10.3390/ijms24054566)
Supplement: Supplementary file 1 [file ijms-24-04566-s001.zip › Supplementary Table S3.pdf]

Supplementary Table S3. Trend analysis of the change in the average number of days with at least ten minutes of leisure time physical activity (LTPA) in general and in intensity categories in relation to optimized polygenetic score (oPGS) values.

	oPGS (0 - 1)<br>n = 116	oPGS (2)<br>n = 223	oPGS (3)<br>n = 210	oPGS (4-5)<br>n = 95	*p* for trend
		Average in MET (95%CI)			
LTPA in general	3.96 (3.17 – 4.76)	4.44 (3.85 – 5.02)	5.10 (4.52 – 5.68)	5.90 (4.95 – 6.84)	0.001**
		Average in MET (95%CI)			*p* for trend
Vigorous	0.75 (0.55 – 0.96)	0.93 (0.76 – 1.10)	0.98 (0.80 – 1.16)	1.35 (1.04 – 1.66)	0.003**
Moderate	1.47 (1.14 – 1.79)	1.53 (1.28 – 1.78)	1.57 (1.32 – 1.82)	2.20 (1.79 – 2.61)	0.014**
Walking	2.19 (1.79 – 2.58)	2.32 (2.02- 2.62)	2.86 (2.56 – 3.17)	2.64 (2.18 – 3.10)	0.009**

*: p <0.05; **: significant results after test correction (p <0.017); 95%CI: 95% confidence interval.
